# Investigating Pathways of Ventilation Induced Brain Injury on Cerebral White Matter Inflammation and Injury After 24 h in Preterm Lambs

**DOI:** 10.3389/fphys.2022.904144

**Published:** 2022-07-04

**Authors:** Kyra YY Chan, Nhi T. Tran, Paris C. Papagianis, Valerie A. Zahra, Ilias Nitsos, Alison M. Moxham, Domenic A. LaRosa, Courtney McDonald, Suzanne L. Miller, Robert Galinsky, Dhafer M. Alahmari, Vanesa Stojanovska, Graeme R. Polglase

**Affiliations:** ^1^ The Ritchie Centre, Hudson Institute of Medical Research, Clayton, VIC, Australia; ^2^ Department of Obstetrics and Gynaecology, Monash University, Clayton, VIC, Australia; ^3^ Monash Biomedicine Discovery Institute and Department of Medical Imaging and Radiation Sciences, Monash University, Clayton, VIC, Australia; ^4^ Monash Biomedical Imaging, Monash University, Clayton, VIC, Australia; ^5^ Department of Diagnostic Imaging, King Saud Medical City, Riyadh, Saudi Arabia

**Keywords:** ventilation-induced brain injury, mechanical ventilation, preterm, white matter injury, mechanisms, cerebral inflammation

## Abstract

Initiation of respiratory support in the delivery room increases the risk and severity of brain injury in preterm neonates through two major pathways: an inflammatory pathway and a haemodynamic pathway. The relative contribution of each pathway on preterm brain injury is not known. We aimed to assess the role of the inflammatory and haemodynamic pathway on ventilation-induced brain injury (VIBI) in the preterm lamb. Fetal lambs (125 ± 1 day gestation) were exteriorised, instrumented and ventilated with a high tidal-volume (V_T_) injurious strategy for 15 min either with placental circulation intact to induce the inflammatory pathway only (INJ_INF_; n = 7) or umbilical cord occluded to induce both the inflammatory and haemodynamic pathways (INJ_INF+HAE_; n = 7). Sham controls were exteriorised but not ventilated (SHAM; n = 5) while unoperated controls (UNOP; n = 7) did not undergo fetal instrumentation. Fetuses were returned *in utero* following intervention and the ewe allowed to recover. Arterial blood gases and plasma were sampled periodically. Twenty-four hours following intervention, lambs were delivered and maintained on non-injurious ventilation for ∼40 min then brains were collected post-mortem for immunohistochemistry and RT-qPCR to assess inflammation, vascular pathology and cell death within white matter regions. Compared to INJ_INF_ lambs, INJ_INF+HAE_ lambs achieved a consistently higher V_T_ during injurious ventilation and carotid blood flow was significantly lower than baseline by the end of ventilation. Throughout the 24 h recovery period, systemic arterial IL-6 levels of INJ_INF+HAE_ lambs were significantly higher than SHAM while there was no difference between INJ_INF_ and SHAM animals. At 24 h, mRNA expression levels of pro-inflammatory cytokines, tight junction proteins, markers of cell death, and histological injury indices of gliosis, blood vessel protein extravasation, oligodendrocyte injury and cell death were not different between groups. Injurious ventilation, irrespective of strategy, did not increase brain inflammation or injury 24 h later when compared to control animals. However, the haemodynamic pathway did influence carotid blood flow adaptations during injurious ventilation and increased systemic arterial IL-6 that may underlie long-term pathology. Future studies are required to further characterise the pathways and their long-term effects on VIBI.

## Introduction

Preterm infants (born <37 completed weeks of gestation) have an increased risk of perinatal brain injury and life-long morbidities, including motor deficits such as cerebral palsy (Chang et al., 2013). Epidemiological studies suggest that the origins of most cases of cerebral palsy are *in utero* and during labour (MacLennan et al., 2015), as well as the initiation of neonatal care procedures such as mechanical ventilation (Graziani et al., 1992; Powell et al., 2006; Drougia et al., 2007). The initiation of positive pressure ventilation (PPV) in the delivery room is sometimes poorly controlled (Schmölzer et al., 2010), with inappropriate high tidal volume ventilation associated with haemorrhagic and diffuse white matter brain injury (Aly et al., 2012; Mian et al., 2019; de Medeiros et al., 2022)—collectively termed ventilation-induced brain injury (VIBI). The major pathological pathways of acute VIBI include cerebral inflammation and haemodynamic instability (Barton et al., 2015a), comparable to the primary etiology of preterm brain injury (Volpe, 2009; Galinsky et al., 2018a).

The inflammatory pathway of VIBI is postulated to be a downstream consequence of ventilation-induced lung injury (Barton et al., 2015a; Chan et al., 2020). In the delivery room, inadvertent excessive tidal volume (V_T_; volutrauma) applied during PPV initiates a pulmonary inflammatory response that can subsequently induce systemic and cerebral inflammation. Indeed, preterm lamb models of injurious mechanical ventilation have reported upregulation of pro-inflammatory cytokine mRNA levels (e.g., interleukin 6 [*IL6*] and *IL8*) and activation of glial cells predominantly within cerebral white matter within 2 h after birth (Polglase et al., 2012; Barton et al., 2015a; Barton et al., 2015b; Barton et al., 2016). An altered cerebral microenvironment and the activation of microglia and astrocytes can contribute to the loss and disturbed maturation of oligodendrocytes, which in turn may contribute to hypomyelination and diffuse white matter injury (Back et al., 2006; Khwaja and Volpe, 2007; Galinsky et al., 2018b).

The haemodynamic pathway of VIBI refers to the irregular variations in pulmonary blood flow caused by loss of cardiac venous return due to removal of the placental circulation at the time of birth, in addition to the high transmural pressures generated inside alveoli during PPV. The increased alveolar transmural pressures results in the compression of pulmonary arterioles, increased pulmonary vascular resistance, destabilisation of left ventricular output and subsequently dysregulated carotid blood flow (CBF) (Polglase et al., 2012; Polglase et al., 2014). Due in part to an underdeveloped cerebral vasculature and immature intrinsic cerebral vasoregulation, the preterm infant may not be able to sustain a stable CBF to compensate for the fluctuating systemic and pulmonary haemodynamic instability (Greisen, 2005; du Plessis, 2009). Cerebral haemodynamic instability, defined as CBF fluctuations longer than 10–20 s, increases the risk of intraventricular haemorrhage which, even at lower grades and in the absence of detectable white matter injury, is associated with poor neurodevelopmental outcomes in preterm infants (Khwaja and Volpe, 2007; Gilmore et al., 2011; Bolisetty et al., 2014).

Despite having identified the two major pathways of VIBI, little is known about the relative contributions of each pathway. Using brain magnetic resonance imaging, we have reported that the haemodynamic pathway has an additive effect on the inflammatory pathway on injury progression following high V_T_ ventilation in preterm lambs (Alahmari et al., 2017). To further those findings, in the current study, we aimed to examine the molecular and immunohistochemical indices of brain injury to further characterise the pathophysiology underpinning the inflammatory and haemodynamic pathways of VIBI in preterm lambs. While previous studies of acute VIBI have only evaluated injury up to 2 h after the initiation of injurious ventilation (Polglase et al., 2012; Barton et al., 2015a; Barton et al., 2015b; Barton et al., 2016), in this study, we aimed to evaluate whether these initial changes in glia population, inflammation and injury persisted or progressed at 24 h. To assess the contribution of the inflammatory and haemodynamic injury pathways to VIBI, we administered injurious ventilation to fetal sheep with either an intact (**INJ**_**INF**_) or occluded placental circulation (**INJ**_**INF+HAE**_). The INJ_INF_ group aimed to isolate predominantly the inflammatory pathway of VIBI, while the transient occlusion of the placental circulation in the INJ_INF+HAE_ group simulated umbilical cord clamping to assess the additive effect of a haemodynamic stressor (i.e., destabilization of left ventricular output) on top of the inflammatory response following high V_T_ ventilation. We hypothesised that white matter inflammation and injury in preterm lambs exposed to both inflammatory and haemodynamic instability pathways of VIBI would be worse than that of lambs exposed to the inflammatory pathway alone.

## Materials and Methods

Experimental procedures were approved by the Monash Medical Centre Animal Ethics Committee A, Monash University, and were conducted in accordance with guidelines established by the National Health and Medical Research Council Code of Practice for the Care and Use of Animals for Scientific Purposes (Eighth Edition). Ewes and lambs in this study were used in a previously published study detailing the experimental model and report the magnetic resonance imaging (MRI) and lung pathology outcomes (Alahmari et al., 2017; Smith et al., 2020). Experimental protocols and outcomes unique to this study are reported in detail below.

Animals were randomly assigned to one of four groups:

1) Unoperated control (**UNOP**; n = 7): Lambs did not undergo fetal surgery or ventilation.
2) Sham surgery control (**SHAM**; n = 5): Lambs underwent fetal surgery, were instrumented and intubated, but did not receive mechanical ventilation.
3) Injurious ventilation (**INJ**_**INF**_; n = 7): Lambs received 15 min of high V_T_ ventilation with an intact placental circulation.
4) Injurious ventilation with umbilical cord occluded (**INJ**_**INF+HAE**_; n = 7): Lambs received 15 min of high V_T_ ventilation during which the umbilical cord was occluded.

### Instrumentation and Injurious Ventilation Strategy

Anaesthesia was induced in pregnant ewes at 125 ± 1 day gestation (mean ± SD; term ∼148 days) by intravenous injection of sodium thiopentone (20 mg/kg; Jurox, NSW, Australia). At this gestation white matter development in the ovine brain is comparable to that of a late preterm human infant (Barlow, 1969; Back and Rosenberg, 2014). Ewes were then intubated with an endotracheal tube (ID 8.0 mm; Smiths Medical, MN, United States) and maintained on an inhalational anaesthetic (isoflurane 1.5%–2.5% in oxygenated air; Bomac Animal Health, NSW, Australia). Under sterile conditions, the head and neck of the fetus were exteriorised for insertion of polyvinyl catheters (ID 0.86 mm, OD 1.52 mm; Dural Plastics & Engineering, NSW, Australia) into the left carotid artery and jugular vein. An ultrasonic flow probe (3PS; Transonic Systems, NY, United States) was placed around the right carotid artery to measure carotid blood flow (CBF); a proxy for cerebral blood flow (Bel et al., 1994). The fetal chest was then exteriorised and the fetus was intubated with a cuffed endotracheal tube (ID 4.0–4.5 mm; Smiths Medical) and lung liquid passively drained for preparation of ventilation. SHAM animals remained intubated and exteriorised for 15 min without mechanical ventilation.

Ventilation was conducted under sterile conditions using a neonatal positive pressure ventilator (Babylog 8,000+, Dräger, Lübeck, Germany) as described previously (Alahmari et al., 2017). Briefly, fetuses (INJ_INF_ and INJ_INF+HAE_) were subjected to positive pressure ventilation for 15 min in volume guarantee with a maximum peak inspiratory pressure set at 40–45 cmH_2_O targeting a tidal volume (V_T_) of 12–15 ml/kg. The 15 min high V_T_ ventilation strategy was conducted to mimic the high V_T_ ventilation that preterm infants often receive during the initial resuscitation in the delivery room (Schmölzer et al., 2010). This tidal volume (V_T_) is known to cause ventilation-induced brain pathology in preterm lambs (Polglase et al., 2012; Skiöld et al., 2014; Barton et al., 2016). INJ_INF+HAE_ animals were simultaneously ventilated with an inflated umbilical cord occluder to remove the placental circulation and simulate the cardiovascular instability associated with umbilical cord clamping (Bhatt et al., 2013). The occluder cuff was deflated and removed at the end of the 15 min injurious ventilation and placental blood flow was restored. The “fetal head-out ventilation” model which involves returning the fetus to the uterus following ventilation was utilized in this study to isolate the effects of ventilation alone, allows for sufficient development of inflammation and injury pathways to manifest into gross lung/brain injury, and perform ventilation strategies at a gestational age in which the sheep’s structural lung development is comparable to that of a very preterm human infant (Alcorn et al., 1981; Chan et al., 2020).

After the 15 min of injurious ventilation, the carotid flow probe was removed, and the fetus was extubated and returned to the uterus. The fetal jugular vein and carotid artery catheters were externalised through the ewe’s right flank to allow access for periodic blood sampling. All incision sites were sutured closed and the ewe and fetus were allowed to recover. Analgesia was provided to the ewe *via* buprenorphine (0.3 mg i. m.; Temgesic; Reckitt Benckiser, United Kingdom) and a transdermal Fentanyl patch (75 μg/h; Janssen-Cilag, NSW, Australia) for post-surgery pain relief. At regular intervals over the subsequent 24 h, fetal arterial blood was sampled for blood gas measurements (ABL80 FLEX, Radiometer Medical ApS, Denmark) to ensure fetal wellbeing and plasma samples were collected for plasma cytokine analysis.

### Lamb Delivery and Subsequent Monitoring

At 24 h post-ventilation, ewes were anaesthetised, the fetus exteriorized, gently ventilated and then delivered as described previously (Alahmari et al., 2017). As part of a concurrent study, methods and results of which have been reported elsewhere (Alahmari et al., 2017), all lambs were placed on a MRI-compatible ventilator (Pneupac^®^ babyPAC™; Smiths Medical, United Kingdom) and MRI images acquired. Lambs were then euthanised (sodium pentobarbitone >100 mg i. v.; Valabarb; Jurox, NSW, Australia) for tissue collection after MRI scanning (total acquisition time 40 min). Ewes were similarly euthanised immediately after delivery of the lambs.

### Brain Collection

At post-mortem examination, the lamb brain was removed from the skull and the cerebrum hemisected along the medial longitudinal fissure. The periventricular and subcortical white matter (PVWM and SCWM respectively) were dissected from the left cerebral hemisphere and snap-frozen in liquid nitrogen. The right cerebral hemisphere was immersion fixed in 10% neutral buffered formalin (NBF; Amber Scientific, WA, Australia) overnight. The right hemisphere was then cut coronally into 5 mm blocks (8–9 blocks/animal), post-fixed in 10% NBF for 6 days, processed, and paraffin-embedded. Serial sections (8 µm) were cut from one block each at the level of frontal, parietal, temporal, and occipital lobes for immunohistochemical analysis.

### Reverse-Transcription Real-Time Quantitative PCR

Frozen brain tissue from the PVWM and SCWM of the left hemisphere were separately homogenised, and RNA extracted from each region (RNeasy Midi RNA Extraction kit; Qiagen, VIC, Australia) and then reverse-transcribed into cDNA following the manufacturer’s instructions (SuperScript^®^ III First-Strand Synthesis System for RT-PCR kit; Invitrogen). Genes of interest relating to key inflammatory interleukins (IL-1 alpha [*IL1A*], IL-1 beta [*IL1B*], IL-6 [*IL6*], tumour necrosis factor alpha [*TNF*]), tight junction proteins (occludin [*OCLN*], claudin 1 [*CLDN1*]), and markers of cell death (p53 [*P53*] and caspase 3 [*CASP3*]) were measured by quantitative PCR using the Fluidigm Biomark HD system (Fluidigm Corporation, CA, United States) (Table 1). Samples were run in quadruplicates and averaged. The expression of all genes were normalised to endogenous housekeeping gene ribosomal protein S18 (*RPS18*) using the 2^–∆∆Ct^ method for each subject and are presented relative to the UNOP group.

**TABLE 1:** Details of genes investigated using RT-qPCR by Fluidigm. The TaqMan assay ID for each probe is provided; target-specific sequences of TaqMan assays are not available due to non-disclosure policies.

Reason for assessment	Gene name	Gene symbol	TaqMan assay ID
References gene	Ribosomal protein S18	*RPS18*	Oa04906333_g1
Cytokines	Interleukin 1 alpha	*IL1A*	Oa04658682_m1
	Interleukin 1 beta	*IL1B*	Oa04656322_m1
	Interleukin 6	*IL6*	Oa04656315_m1
	Tumour necrosis factor alpha	*TNF*	Oa04655425_g1
Tight junction proteins	Claudin 1	*CLDN1*	Oa03217991_m1
	Occludin	*OCLN*	Oa04728970_m1
Markers of cell death	Tumor protein p53	*P53*	Oa03223218_g1
	Caspase 3	*CASP3*	Oa04817361_m1

Gene symbols for corresponding genes are italicised.

### Immunohistochemistry

Coronal sections were stained with rabbit anti-ionised calcium binding adapter molecule-1 (Iba-1; 1:1,500, Wako Pure Chemical Industries, Osaka, Japan) to identify microglia; rabbit anti-sheep serum (1:1,000, Sigma-Aldrich, MO, United States) to identify vascular extravasation of protein; mouse anti-oligodendrocyte transcription factor-2 (Olig2; 1:1,000, Merck, Darmstadt, Germany) for oligodendrocytes; rat anti-myelin basic protein (MBP; 1:200; Merck, Darmstadt, Germany) for mature myelin and oligodendrocytes; and rabbit anti-glial fibrillary acidic protein (GFAP; 1:400, Sigma-Aldrich, MA, United States) to identify astrocytes. Prior to incubation with primary antibodies, antigen retrieval was conducted by heating in citrate buffer (10 mM Tri-sodium citrate in dH_2_O, pH 6.0; Sigma Aldrich) in a microwave oven. Antigen retrieval was not conducted for sheep serum staining. Sections were blocked for non-specific binding with Dako blocking reagent (Dako, United States) for 1 h prior to overnight incubation (at 4°C) in primary antibodies. Sections then incubated with secondary biotinylated IgG antibody raised in the corresponding animal (1:200; Vector Laboratories, CA, United States) then reacted using the Vectastain Elite ABC Kit (Vector Laboratories, CA, United States). In addition, a ‘Terminal deoxynucleotidyl transferase dUTP nick end labelling’ (TUNEL) assay (ApopTag^®^ Peroxidase *In Situ* Apoptosis Detection Kit, Millipore, United States) was conducted according to manufacturer’s instructions. Sections reacted with anti-sheep serum, TUNEL and Olig2 were counterstained with 20% haematoxylin. Staining was absent when the primary antibody was omitted.

### Quantitative Immunohistochemical Analysis

Analyses were conducted at equivalent sites within the cerebral white matter (PVWM and SCWM) of sections from the frontal, parietal, temporal, and occipital lobes of each lamb (i.e., 4 sections per lamb). Slides were scanned by Monash Histology using an eSlide capturing device (Aperio Scanscope AT Turbo; Aperio Technologies) to allow for quantitative assessments. For manual quantification of Iba-1- (total cells and ameboid microglia), GFAP-, TUNEL-, Olig2-, MBP-positive areal density (cells/mm^2^) and Iba-1, GFAP- and MBP-positive staining fraction areal coverage (%), 3 non-overlapping fields of view in the PVWM were taken medial to lateral from the ventricle, and 6 non-overlapping fields in the SCWM were obtained from alternating gyri starting from the second gyrus closest to the midline were assessed on each slide (Field of view (FOV) area = 0.14 mm^2^; total of 12 PVWM and 24 SCWM FOVs across the brain). For Iba-1 analysis, positive staining within cell bodies with and without processes were identified as an individual microglia cell and ameboid microglial were identified by characteristic round, densely stained Soma with resorbed processes (Kettenmann et al., 2011). Microglial aggregations were identified as dense clusters of positive staining and the number of aggregation populations within white matter regions were counted. The total number of blood vessel profiles with protein (sheep serum) extravasation and total areal density TUNEL-positive cells were manually quantified in entire white matter regions of each lobe. All FOV were assessed and then an average calculated across all FOV and lobes. Slides were coded and the observers (KC & NTT) were blinded to the treatment. Analysis for areal density counts were conducted using ImageScope (Aperio Technologies; Leica Biosystems, Germany). For assessments of fractional areal coverage, FOV areas were exported from ImageScope and imported to ImageJ software (FIJI, National Institutes of Health, United States), and an optimised set threshold was used to calculate area coverage using ImageJ. The set threshold was optimised by calculating an average threshold that would allow optimal detection of positive staining in FOVs, the optimised threshold was then set for all slide assessments.

### Plasma Protein Analysis

Arterial blood was collected *via* the fetal carotid artery catheter before injurious ventilation (Time = 0), at the end of injurious ventilation (15 min), and post-surgery until delivery (1, 3, 6, 12, 24 h) for assessment of plasma proteins (IL-6 and IL-8) using a sandwich enzyme-linked immunosorbent assay (ELISA) assay as described previously (Kelly et al., 2021). Plates were read on a SpectraMax i3 microplate reader (Molecular Devices, CA, United States) at 450 nm to determine optical density (OD). Standards (recombinant ovine IL-6 or IL-8; Kingfisher Biotech, MN, United States) were included and a standard curve was generated for every ELISA plate used (*R* ^2^ > 0.99).

### Statistical Analysis

Data were tested for normality by Shapiro-Wilk test and statistical analysis conducted accordingly. Data violating the assumption of sphericity was corrected with the Greenhouse-Geisser method. Statistical significance was accepted at *p* < 0.05. A mixed-effects restricted maximum likelihood (REML) model was applied to blood gas, V_T_, carotid blood flow and ELISA data. The independent variables assessed were group (P_GROUP_) and time of measurement (P_TIME_). Where there was a significant interaction between independent variables (P_GROUP X TIME_), a post-hoc analysis with Tukey’s multiple comparisons test was undertaken. Significant P_TIME_ was followed up by comparisons to baseline values. Fetal parameters, RT-qPCR (log-transformed data), and immunohistochemical data were compared using one-way analysis of variance with Tukey post hoc comparison where significance was revealed. Statistical analyses were conducted using GraphPad Prism (version 8.1.2; GraphPad Software, CA, United States). Data are presented as mean ± SD, unless otherwise stated.

## Results

### Blood-Gas Parameters

**FIGURE 1:**
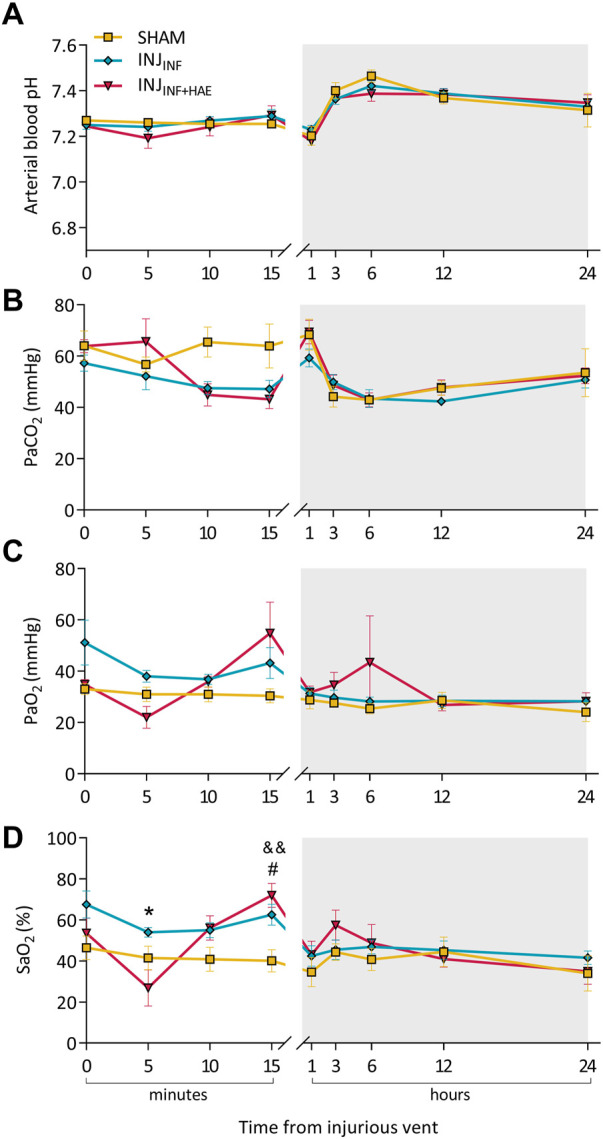
Arterial blood gas parameters during the injurious ventilation and recovery period (grey bar). **(A)** pH, **(B)** partial pressure of carbon dioxide (PaCO_2_), **(C)** partial pressure of oxygen (PaO_2_), and **(D)** oxygen saturation level (SaO_2_) at specific timepoints after commencement of injurious ventilation of SHAM (yellow), INJ_INF_ (blue), and INJ_INF+HAE_ (red) animals. #*p* < 0.05 SHAM vs. INJ_INF_; ^&&^ *p* < 0.01 SHAM vs. INJ_INF+HAE_; *p < 0.05 INJ_INF_ vs. INJ_INF+HAE_. Data presented as mean ± SEM.

During the injurious ventilation and 24 h recovery period, pH and PaO_2_ were not different between groups (Figures 1A–C). There were significant interactions of time and group for PaCO_2_ (P_GROUP X TIME_ = 0.017) and SaO_2_ (P_GROUP X TIME_ = 0.0001) (Figures 1B–D). At 5 min of injurious ventilation, SaO_2_ was significantly reduced in the INJ_INF+HAE_ group compared to INJ_INF_ (*p* = 0.049) but returned to similar levels by 15 min, at which time both ventilation groups were significantly higher than SHAM (INJ_INF_: *p* = 0.035 and INJ_INF+HAE_: *p* = 0.007).

There were no differences in blood gas parameters during the recovery period, and at 24 h post injurious ventilation, blood gas parameters as well as lamb body and brain weights recorded at post-mortem were not different between groups (Table 2).

**TABLE 2:** Lamb characteristics and fetal arterial blood gas parameters at the end of the experiment (24 h post injurious ventilation). Data are presented as mean ± SD.

	UNOP	SHAM	INJ_INF_	INJ_INF+HAE_
**Group characteristics**				
Number (n)	7	5	7	7
Gestational age (days)	126 ± 1	126 ± 1	126 ± 1	126 ± 1
Sex (% male)	85.7	60.0	71.4	14.3
Birth order 1st (ratio)	6:1	4:1	7:0	7:0
Body weight (kg)	3.42 ± 0.42	3.31 ± 0.25	3.36 ± 0.46	3.17 ± 0.39
Brain weight (g)	45.61 ± 2.62	45.99 ± 2.49	46.34 ± 2.42	45.90 ± 3.20
**Arterial blood gas parameters**				
pH	7.27 ± 0.04	7.31 ± 0.07	7.33 ± 0.07	7.35 ± 0.09
PaCO_2_ (mmHg)	59.02 ± 7.82	53.50 ± 9.37	50.74 ± 8.29	52.31 ± 8.21
PaO_2_ (mmHg)	29.80 ± 6.30	24.00 ± 3.63	28.29 ± 3.25	28.29 ± 8.75
SaO_2_ (%)	48.17 ± 23.09	33.90 ± 19.21	41.56 ± 8.74	34.87 ± 16.49

*PaCO*_*2*_ partial pressure of carbon dioxide; *PaO*_*2*_ partial pressure of oxygen; *SaO*_*2*_ oxygen saturation.

### Injurious Ventilation and Carotid Blood Flow

**FIGURE 2:**
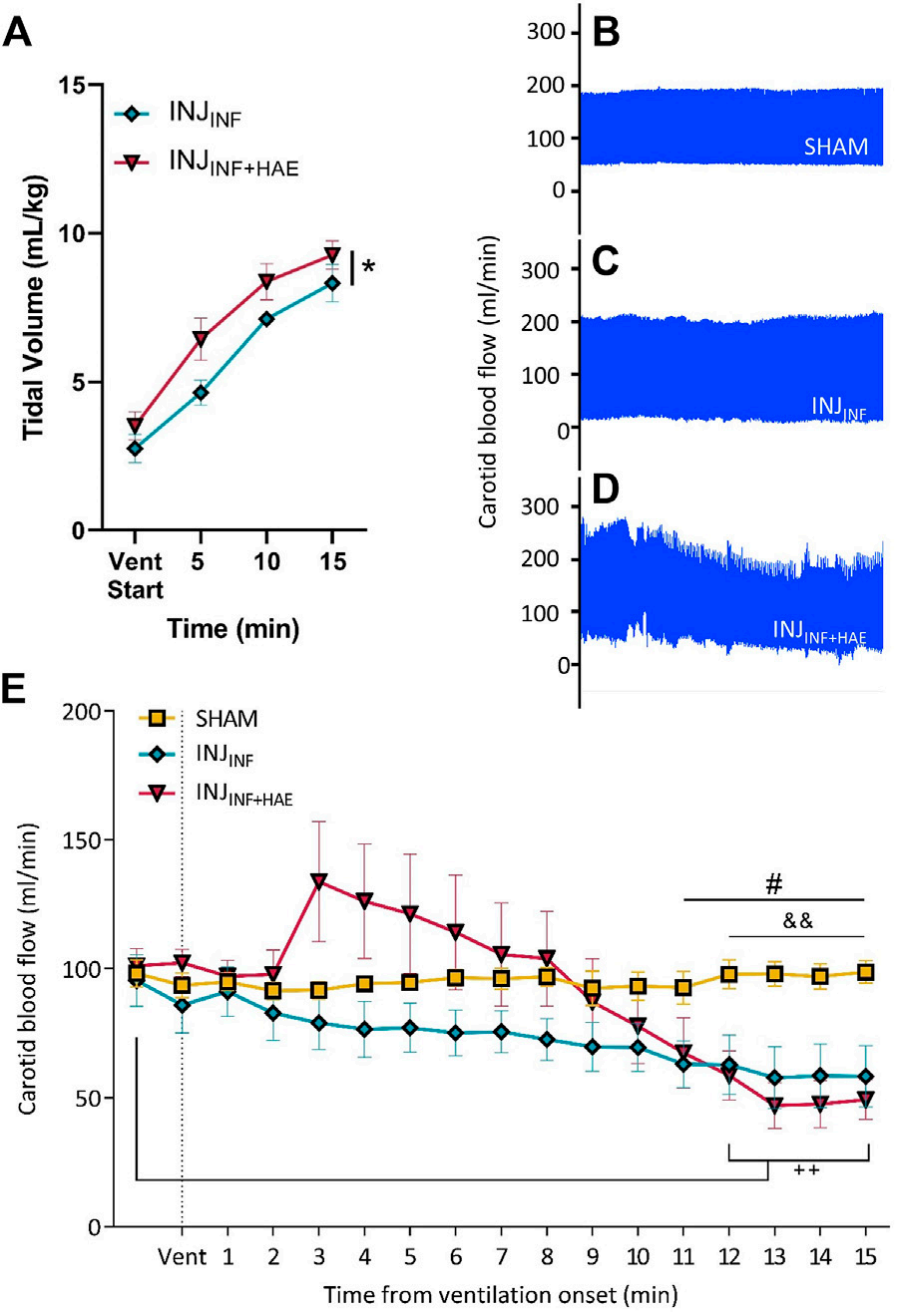
**(A)** Tidal volume (V_T_) increased over time in INJ_INF_ (blue) and INJ_INF+HAE_ (red) animals during injurious ventilation. V_T_ was higher in INJ_INF+HAE_ than INJ_INF_; *p < 0.05 INJ_INF_ vs. INJ_INF+HAE_. **(B–D)** Representative snapshots of carotid blood flow (CBF) where INJ_INF+HAE_ animals that received injurious ventilation with umbilical cord occlusion showed the greatest CBF instability. **(E)** CBF during injurious ventilation. Dotted line indicates when injurious ventilation commenced. Data presented as mean ± SEM. #*p* < 0.05 SHAM vs. INJ_INF_; ^&&^ *p* < 0.01 SHAM vs. INJ_INF+HAE_; ^++^ *p*< 0.01 INJ_INF+HAE_ vs. baseline.

V_T_ was significantly higher in the INJ_INF+HAE_ group compared to the INJ_INF_ group (P_GROUP_ = 0.046). However, in both groups, V_T_ failed to reach the target of 12–15 ml/kg (INJ_INF_: 8.32 ± 1.67 ml/kg vs. INJ_INF+HAE_: 9.26 ± 1.27 ml/kg; Figure 2A).

CBF was stable in the SHAM group over the 15 min period the lambs were exteriorised but not ventilated (coefficient of variation = 2.5%, Figure 2B). Compared to SHAM animals, CBF was more variable in INJ_INF_ animals (coefficient of variation = 15.8%; Figure 2C) and most variable in INJ_INF+HAE_ lambs (coefficient of variation = 30.5%; Figure 2D). INJ_INF+HAE_ lambs had significantly lower CBF than baseline (pre-ventilation) at 12–15 min (*p* < 0.01; Figure 2E). When comparing INJ_INF_ and INJ_INF+HAE_ animals, there remained a significant effect of the haemodynamic response on CBF over the 15 min of ventilation (P_GROUP X TIME_<0.0001) however post-hoc tests revealed no differences at any timepoint. CBF was significantly lower in both ventilation groups by 11–12 min compared to SHAM lambs (*p* = 0.003–0.043; Figure 2E).

### Effect of Ventilation on Plasma Cytokine Levels

**FIGURE 3:**
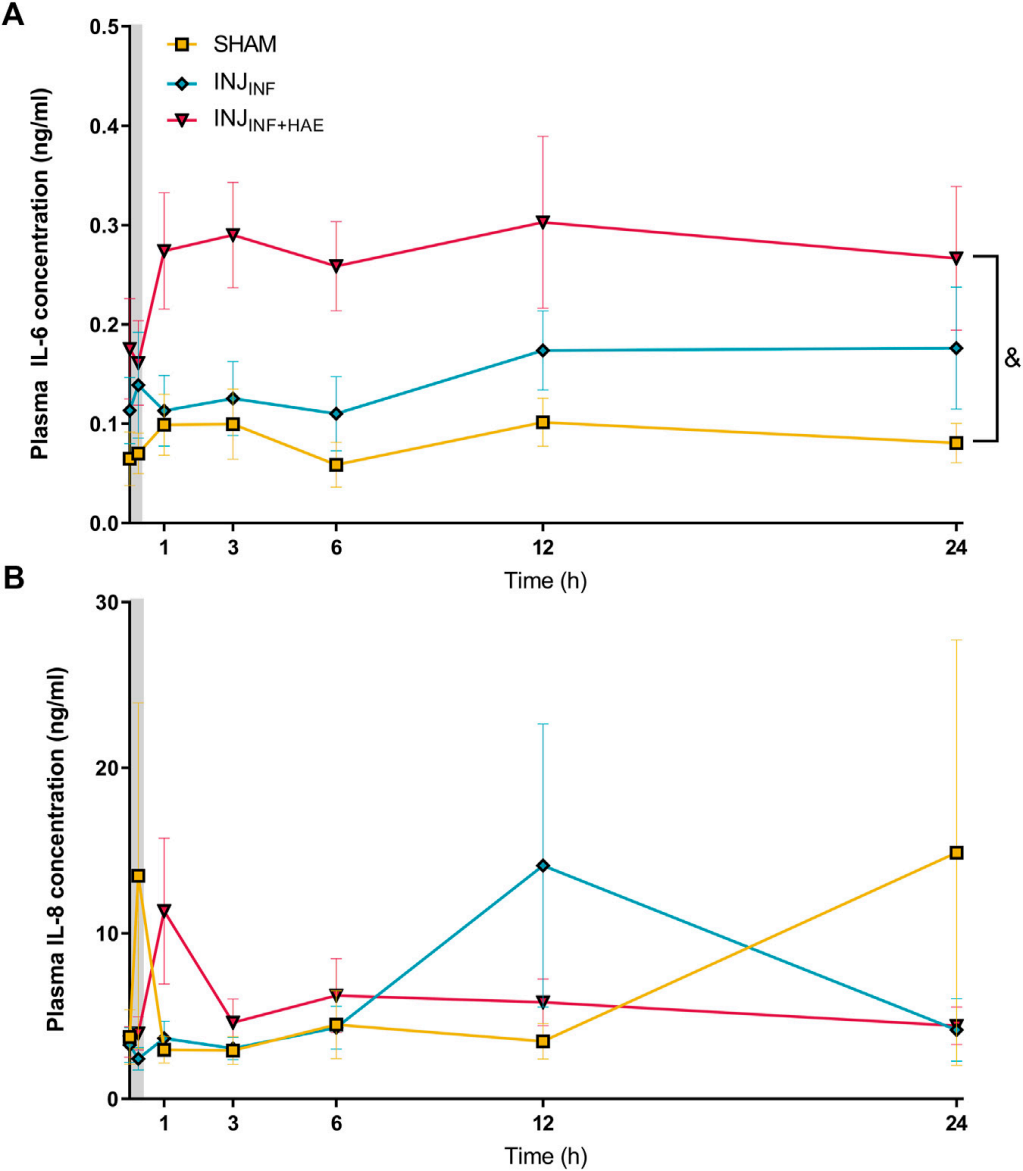
Plasma levels of pro-inflammatory cytokines **(A)** interleukin (IL)-6 and **(B)** IL-8 after injurious ventilation in SHAM (yellow), INJ_INF_ (blue), and INJ_INF+HAE_ (red) animals. Grey bar denotes injurious ventilation duration. Data are not available for unoperated controls as plasma was not obtained. ^&^ *p* < 0.05 SHAM vs. INJ_INF+HAE_. Data presented as mean ± SEM.

Plasma IL-6 levels in INJ_INF+HAE_ were significantly elevated compared to the SHAM lambs (*p* = 0.020) but there was no difference between INJ_INF_ and SHAM lambs (*p* = 0.099; Figure 3A). Plasma IL-8 levels did not change over time and were not significantly different between groups (P_GROUP X TIME_ = 0.057) (Figure 3B).

### Gene Expressions Levels in PVWM and SCWM

**FIGURE 4:**
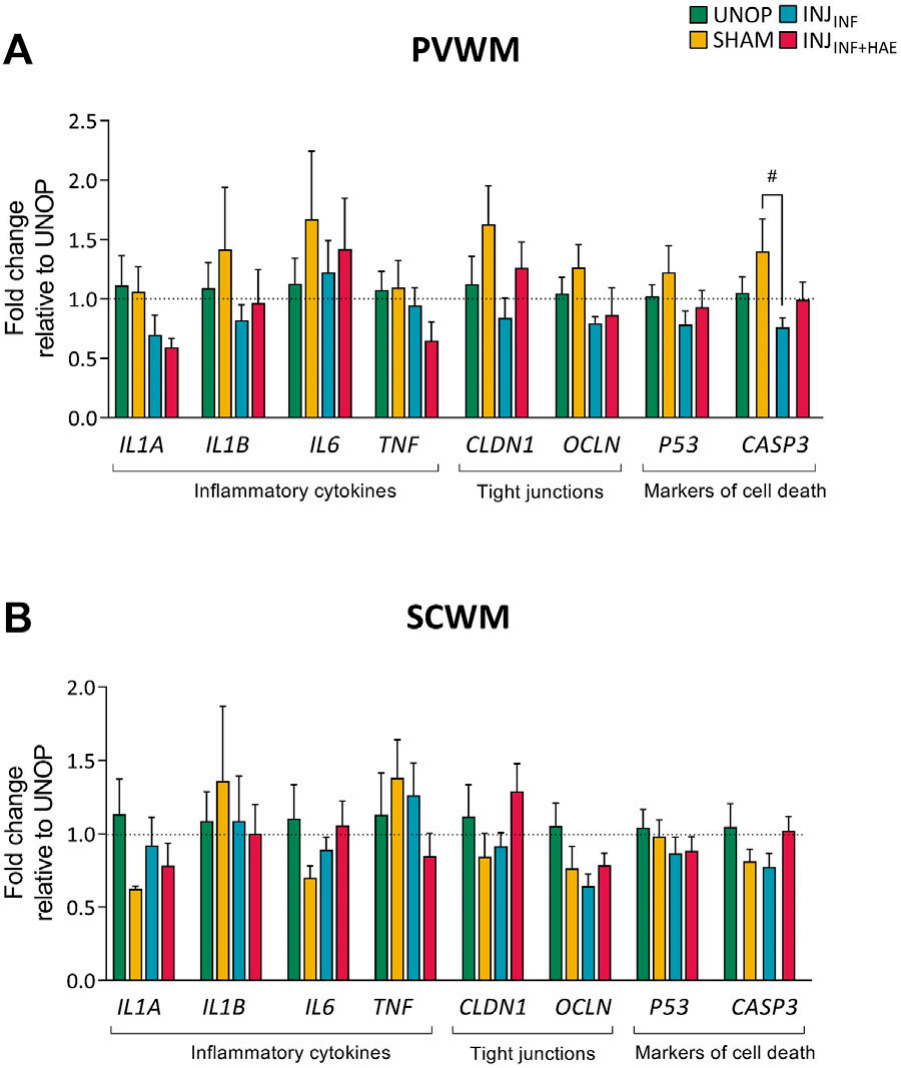
Gene expression levels of inflammatory cytokines (interleukin 1 alpha [*IL1A*], interleukin 1 beta [*IL1B*], interleukin 6 [*IL6*], tumour necrosis factor [*TNF*]), tight junction proteins (claudin 1 [*CLDN1*], occludin [*OCLN*]), and markers of cell death (p53 [*P53*], caspase 3 [*CASP3*]) in the **(A)** PVWM and **(B)** SCWM were not different between groups. Data presented as mean ± SEM.

There was a significant decrease in mRNA levels of *CASP3* in INJ_INF_ lambs within the PVWM compared to SHAM lambs (*p* = 0.034; Figure 4A). mRNA levels of inflammatory related cytokines *IL1A*, *IL1B*, *IL6*, and *TNF*, tight junction proteins *CLDN1* and *OCLN* and cell death markers *P53* in the PVWM and SCWM were not different between groups (Figures 4A,B).

### Immunohistochemical Assessment of Brain Injury

**FIGURE 5:**
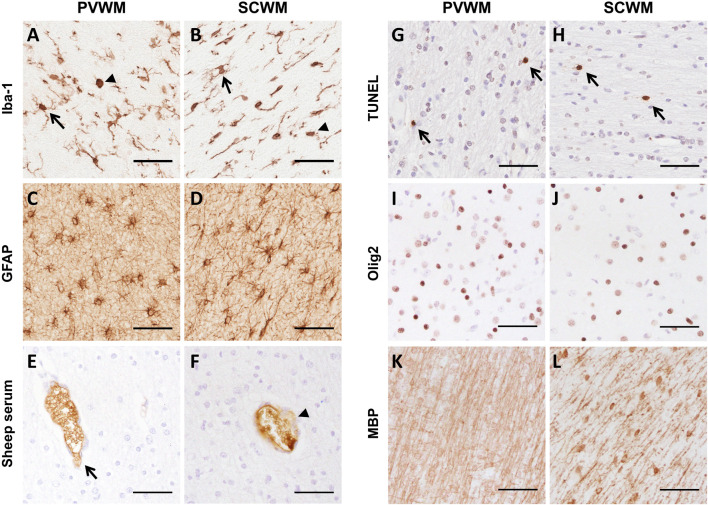
Representative images of immunohistochemical staining of **(A,B)** Iba-1 (arrows indicate ramified microglia and arrowhead indicates amoeboid microglia), **(C,D)** GFAP, **(E,F)** sheep serum (arrow indicates intact vessel, arrowhead indicates vessel profile with protein extravasation), **(G,H)** TUNEL (arrows indicate positive staining), **(I,J)** Olig2 and **(K,L)** MBP in the PVWM and SCWM. Scale bars = 50 µm.

Changes to microglia and astrocytes that indicate a neuroinflammatory state were assessed using antibodies specific for microglia (Iba-1) and astrocytes (GFAP). There were no changes in the areal density and area coverage of Iba-1-positive stained microglia, the percentage of amoeboid microglia, the number of Iba-1-positive aggregations and the area occupied by these aggregations within the PVWM and SCWM between groups (all *p* > 0.05; Table 3 and Figures 5A,B). Astrocyte accumulation, as indicated by the density of GFAP-positive cells and the area coverage of GFAP-positive staining, did not differ between groups in the PVWM and SCWM (all *p* > 0.05; Table 3 and Figures 5C,D).

**TABLE 3:** Summarised outcomes of immuno-positive staining assessed in unoperated controls (UNOP; n = 7), sham (SHAM; n = 5), injurious ventilation with (INJ_INF_; n = 7) and without (INJ_INF+HAE_; n = 7) placental circulation. Data shown as mean ± SD.

	PVWM					SCWM			
	UNOP	SHAM	INJ_INF_	INJ_INF+HAE_	UNOP	SHAM	INJ_INF_	INJ_INF+HAE_
Iba-1									
Areal density (cells/mm^2^)	114.97 ± 15.17	117.67 ± 7.84	111.71 ± 19.56	120.51 ± 24.10	111.42 ± 8.85	100.63 ± 9.45	102.88 ± 7.71	110.20 ± 13.50
Area coverage (%)	3.10 ± 3.49	4.21 ± 3.40	2.26 ± 1.68	2.01 ± 1.24	6.20 ± 3.78	4.73 ± 3.16	2.41 ± 1.57	4.82 ± 3.05
Percentage of ameboid micoglia (%)	6.25 ± 2.92	5.25 ± 1.16	6.52 ± 2.36	6.97 ± 1.12	5.00 ± 1.23	5.91 ± 1.71	5.19 ± 2.02	5.01 ± 1.90
Number of microglial aggregations (n)	0.33 ± 0.33	0.47 ± 0.18	0.38 ± 0.30	0.48 ± 0.26	1.68 ± 1.20	0.88 ± 1.00	1.07 ± 0.81	1.29 ± 0.94
GFAP									
Area coverage (%)	8.61 ± 1.58	7.44 ± 3.13	8.11 ± 2.01	6.58 ± 2.31	8.36 ± 1.28	6.88 ± 1.57	7.96 ± 2.14	6.80 ± 2.20
Areal density (cells/mm^2^)	39.24 ± 9.26	40.59 ± 8.38	37.33 ± 5.02	36.37 ± 6.46	45.16 ± 8.21	44.58 ± 9.89	46.95 ± 8.58	43.25 ± 10.17
Sheep serum									
Number of extravasation (n)	2.25 ± 1.24	2.43 ± 1.42	1.91 ± 1.03	2.57 ± 1.15	9.79 ± 4.56	7.10 ± 2.05	11.04 ± 6.18	10.76 ± 4.38
TUNEL									
Areal density (cells/mm^2^)	1.79 ± 1.05	1.59 ± 1.37	1.61 ± 0.98	1.77 ± 0.76	10.24 ± 9.04	5.36 ± 6.54	5.63 ± 3.19	5.42 ± 2.14
Olig2									
Areal density (cells/mm^2^)	537.44 ± 77.43	575.60 ± 102.80	562.36 ± 143.10	526.12 ± 73.08	532.11 ± 88.23	531.78 ± 87.42	561.56 ± 162.60	492.14 ± 60.44
MBP									
Area coverage (%)	23.37 ± 5.12	24.41 ± 5.82	29.45 ± 4.43	27.98 ± 3.00	21.91 ± 3.25	22.43 ± 7.08	25.06 ± 3.74	26.40 ± 1.74

There was no difference in the number of blood vessel profiles with protein extravasation between groups, indicating similar vascular integrity (all *p* > 0.05; Table 3 and Figures 5E,F). ApopTag immunohistochemistry revealed no increases in cell death following ventilation (all *p* > 0.05; Table 3 and Figures 5G,H).

Total numbers of (Olig-2 positive) oligodendrocytes as well as area coverage of myelin (MBP positive staining) did not differ between groups (all *p* > 0.05; Table 3 and Figures 5I–L).

## Discussion

Preterm neonates exposed to high tidal volumes in the delivery room are at increased risk of brain inflammation and injury, but the aetiology and the relative contribution of the inflammatory and haemodynamic pathways to VIBI are not well understood. In this study, we found that INJ_INF+HAE_ lambs achieved a consistently higher V_T_ during injurious ventilation which were significantly more likely to experience fluctuations in carotid blood flow characteristic of haemodynamic instability. While we observed an increase in systemic IL-6 in INJ_INF+HAE_ lambs during the 24 h recovery period, at 24 h, we found no overt molecular and histopathological indices of white matter brain inflammation and injury after injurious ventilation in either of the injuriously ventilated groups.

Whilst we did not reach the target V_T_ of 12–15 ml/kg, the achieved V_T_ of 7–10 ml/kg is higher than the recommended V_T_ of 5–7 ml/kg for lambs of this gestational age (Probyn et al., 2005; Hillman et al., 2007; Polglase et al., 2012; Nott et al., 2020). The inability to reach the intended high V_T_ despite volume-guaranteed ventilation highlights the immaturity of the preterm lamb lungs at this gestation, which have very low compliance and high airway resistance. Notwithstanding this, INJ_INF+HAE_ lambs achieved a consistently and significantly higher V_T_ (∼23% higher) compared to the INJ_INF_ group during the 15 min of high V_T_ ventilation. Interestingly, a similar effect of an intact placental circulation on V_T_ was observed in lambs exposed to intrauterine inflammation, where V_T_ was significantly higher in immediate cord clamping lambs compared to physiological-based cord clamping (initiating respiratory support prior to umbilical cord clamping) (Lio et al., 2018). The reason for the lungs ability to obtain higher VTs when the umbilical cord is occluded is not known. Occluding the umbilical cord simulates immediate cord clamping and removal of the placental circulation at birth. Ventilation after umbilical cord clamping results in a rapid increase in pulmonary blood flow and decrease in pulmonary vascular resistance (Bhatt et al., 2013). A subsequent overall decrease in pulmonary interstitial tissue pressure in the INJ_INF+HAE_ lambs may therefore allow for greater V_T_ to be achieved at the same pressures. Another potential mechanism for the increased tidal volumes is the additional need to maintain haemodynamic stability to promote pulmonary blood flow to decrease pulmonary resistance (Lakshminrusimha and Steinhorn, 1999). Future studies are required to elucidate the exact mechanisms underlying increased tidal volume following ventilation umbilical cord clamping.

Immediate cord clamping also results in fluctuations in blood pressure and carotid blood flow during the initial 15 min of ventilation (Bhatt et al., 2014). Accordingly, the initiation of the haemodynamic transition caused by transient occlusion of the umbilical cord in the INJ_INF+HAE_ group also increased variability of CBF, the biphasic response in SaO_2_ and the higher fraction of inspired oxygen requirement, as reported elsewhere (Alahmari et al., 2017). Fluctuations in CBF increases the risk of intraventricular haemorrhage (Perlman et al., 1983; Calvert et al., 1988). Despite the demonstrated effects on CBF in INJ_INF+HAE_ lambs, this did not translate into increased cerebral vascular leakage as has been shown previously (Polglase et al., 2012). Conversely, CBF remained stable in the INJ_INF_ lambs and gradually decreased over the 15 min of ventilation, consistent with the pattern of change in CBF observed in human preterm infants after birth (Gleason et al., 1988; Winberg et al., 1990). Previous studies have shown that preventing CBF fluctuations at birth reduces adverse outcomes (Perlman et al., 1985; Polglase et al., 2012) which highlights the importance of targeting the haemodynamic pathway of VIBI to improve neonatal outcomes.

Interestingly, systemic arterial IL-6 levels were significantly elevated in the INJ_INF+HAE_ lambs up to 24 h following injurious ventilation but not in the INJ_INF_ lambs. This indicates that occlusion of the umbilical cord in combination with injurious respiratory support can amplify the systemic inflammatory cascade. Lambs that did not experience haemodynamic instability did not demonstrate an increase in systemic arterial IL-6 levels, further supporting that haemodynamic instability is an important mediator of ventilation-related injury. However, despite an increase in circulating IL-6 in the INJ_INF+HAE_ lambs, there was no concordant increase in mRNA expression of IL-6 and other inflammatory cytokines nor histological evidence of increased cerebral inflammation. We did not evaluate cerebral inflammation at earlier time points; therefore, the lack of cerebral inflammatory markers at 24 h could be due to either a temporal resolution of inflammation or the absence of cerebral inflammation. The former hypothesis is consistent with a previous study demonstrating that inflammatory cytokines are maximally increased within 1–6 h after an injurious 15 min of high V_T_ ventilation, which is then resolved by 24 h (Hillman et al., 2011). Conversely, the biotrauma of ventilation may have not been severe enough to trigger severe inflammatory signalling that would potentiate cerebral inflammation (Kempuraj et al., 2017; Witzenrath and Kuebler, 2021). This may be the case in the INJ_INF+HAE_ lambs despite the increase in systemic arterial IL-6 concentrations. The increase in systemic IL-6 levels may have reflected a buffering role for immune monitoring given its dual role in pro-inflammatory and anti-inflammatory signalling (Turner et al., 2014). Indeed, a recent study in overventilated mice suggested a capacity for systemic IL-6 signalling to cross the blood-brain barrier (BBB) and induce neuronal injury (Sparrow et al., 2021). Furthermore, previous studies in preterm fetal sheep have demonstrated a strong association between increased circulating IL-6 levels and neuroinflammation and injury (Galinsky et al., 2020a; Galinsky et al., 2020b). However, IL-6 levels were substantially higher (10–100-fold higher) compared to the levels observed in our study, indicating that the magnitude of the increase in IL-6 achieved in this study was insufficient to induce markers of inflammation and injury in the brain.

We did not observe white matter injury in any brains when examined at 24 h after injurious ventilation. There was no sign of disturbed cerebral vascular integrity nor increased glial cell accumulation at 24 h which could be due to either a temporal resolution or a complete lack of neuroinflammation and BBB disturbance. For example, alterations to the BBB and tight junction protein redistribution and remodelling can occur rapidly (within minutes to hours after insult) (Stonestreet et al., 2000; Winger et al., 2014), while glial cell accumulation varies temporally with injury severity (Malaeb and Dammann, 2009). White matter damage or loss of myelination is a well-established characteristic of preterm brain injury, and the risk of white matter injury can be further exacerbated by injurious ventilation (Khwaja and Volpe, 2008; Barton et al., 2015a). We have previously reported lower axial and radial diffusivity particularly in the frontal white matter in the same ventilated lambs (Alahmari et al., 2017), suggesting a histopathological effect of injurious ventilation was identifiable 24 h after the insult. These magnetic resonance imaging data are in contrast to the lack of changes to oligodendrocyte density and myelin coverage found using immunohistochemical techniques observed in this study. It is worth noting however that MBP immunohistochemical staining does not delineate intact myelin from myelin debris, nor inform alterations to directionality of fibers or axonal water content (Budde et al., 2009). Nonetheless, given the putative role of caspase-3 in cell death, the small decrease in *CASP3* mRNA in the PVWM in the absence of increased histochemical markers of cell death does suggest white matter disturbances (Truttmann et al., 2020). Moreover, the reduction in brain metabolites creatine and choline observed *via* magnetic resonance spectroscopy reported previously in these preterm ventilated lambs (Alahmari et al., 2017), as well as the physiological changes observed in this study, suggests a potential disturbance to cerebral metabolism which may ultimately contribute to neuronal dysfunction (Blüml et al., 2014).

The experimental design used in this study was intended to isolate the consequences of the initial volutrauma from any subsequent inflammation and injury by returning placental circulation after ventilation (Hillman et al., 2007; Chan et al., 2020). Other preterm lamb studies that utilised the same injurious ventilation strategy (15 min of high V_T_ ventilation) have reported detectable brain injury by ∼2 h (i.e., increased mRNA expression of pro-inflammatory cytokines, compromised BBB, increased microgliosis, astrogliosis, and markers of cell death in the PVWM and SCWM) (Polglase et al., 2012; Barton et al., 2015b; Barton et al., 2016). In these studies however, ventilation was maintained for up to 2 h after the initial 15 min injurious ventilation using a gentle ventilation strategy, demonstrating that even when gentle or protective ventilation strategies are used, the immature brain remains at high risk of injury. Indeed, it is well established that the duration of mechanical ventilation increases the risk for white matter injury including periventricular leukomalacia in human newborns (Gagliardi et al., 2009). In fact, 2 h of mechanical ventilation significantly increases the levels of multiple pro-inflammatory cytokines (IL-8, IL-1β, TNF) and reduces anti-inflammatory IL-10 levels in newborn arterial blood (Bohrer et al., 2010); and can persist with prolonged ventilation (Bose et al., 2013). Given the correlation between inflammatory cytokines and increased risk of preterm brain damage (McAdams and Juul, 2012), it is therefore not surprising we were unable to detect severe brain injury in this particular ventilation paradigm that only applied 15 min of injurious ventilation. Moreover, this study used *in utero* post-injurious ventilation recovery and not *ex utero* recovery as used in the aforementioned studies (i.e., lambs were delivered prior to volutrauma). Indeed, the influence of placental blood flow and ductal shunting in reducing lung injury has been shown previously (Hillman et al., 2007). Therefore it would not be surprising to see similar effects on the brain in terms of inflammation and injury given the fetus’ capacity for brain sparing during periods of pathophysiology (Giussani, 2016) and the role the placenta has in regulating fetal pulmonary circulation and oxidative stress responses (Rees et al., 2011; Cannavò et al., 2020). The results of previous studies (Polglase et al., 2012; Barton et al., 2015b; Barton et al., 2016) and this study collectively suggest that an initial 15 min of high V_T_ ventilation alone is not sufficient to cause brain injury, but rather it is the additional maintenance of mechanical ventilation that precipitates ventilation-induced brain injury. Studies are currently underway to test this hypothesis in preterm lamb models of continuing mechanical ventilation *in utero* to ascertain the relative contributions of the inflammatory and haemodynamic response pathway in VIBI.

The *in utero* experimental design does however provide unique advantages over those conducted *ex utero* as previously discussed in our recent review (Chan et al., 2020). The neonatal ventilation model (i.e., lamb is delivered then ventilated), while more reflective of clinical care, is limited to the maturity of the lungs. For instance, at 125 days of gestation where term is 148 days, the sheep lung is comparable to a 26–28 weeks human infant (Alcorn et al., 1981; Schittny, 2017), while the sheep white matter is equivalent to a late preterm fetus (Barlow, 1969). Therefore, studies of ventilation can only be maintained for ∼24 h until the risk of adverse effects caused by ventilation itself such as pulmonary vascular dysfunction and pneumothoraces rapidly increases, thereby introducing multiple confounding factors to assessments of the brain in isolation (Malhotra et al., 2018). In addition, the *in utero* experimental design is void of the confounding effects of antenatal glucocorticoids (Miller et al., 2012), anaesthesia (Lee et al., 2015) and temperature (Jia et al., 2013) on the brain. Indeed, future studies investigating longer durations of ventilation using this experimental model will provide mechanistic evidence for ventilation strategies like physiological-based cord clamping, especially in terms of the brain.

## Conclusion

In this study, we set out to examine whether the haemodynamic pathway of injury has additive effects to the inflammatory pathway on the progression of VIBI. We demonstrate that additive haemodynamic instability induces greater fluctuations in CBF during ventilation that resulted in persistently increased systemic IL-6 levels. Despite the absence of significant cerebral white matter damage at 24 h following 15 min of ventilation with V_T_ of ∼7–10 ml/kg irrespective of intact placental circulation, our results suggest that reducing the influence of the haemodynamic pathway of brain injury, using strategies like physiological-based cord clamping (Bhatt et al., 2014; Polglase et al., 2018) may improve injurious physiological responses to ventilation. Further studies are needed to characterise the effects of prolonged ventilation on the immature brain of preterm infants.

## Data Availability

The original contributions presented in the study are included in the article/supplementary material, further inquiries can be directed to the corresponding author.
